# Quantitative EEG reactivity induced by electrical stimulation predicts good outcome in comatose patients after cardiac arrest

**DOI:** 10.1186/s13613-024-01339-6

**Published:** 2024-06-27

**Authors:** Gang Liu, Yuan Wang, Fei Tian, Weibi Chen, Lili Cui, Mengdi Jiang, Yan Zhang, Keming Gao, Yingying Su, Hongxing Wang

**Affiliations:** 1 Department of Neurology, Xuanwu Hospital, Capital Medical University, National Brain Injury Evaluation Quality Control Center, National Center for Neurological Disorders, National Clinical Research Center for Geriatric Diseases, Beijing, 100053 China; 2 Department of Psychiatry, Mood Disorders Program, University Hospitals Cleveland Medical Center/Case Western Reserve University, Cleveland, OH 44106 USA

**Keywords:** EEG reactivity, Cardiac arrest, Coma, Prognostication

## Abstract

**Background:**

EEG reactivity is a predictor for neurological outcome in comatose patients after cardiac arrest (CA); however, its application is limited by variability in stimulus types and visual assessment. We aimed to evaluate the prognostic value of the quantitative analysis of EEG reactivity induced by standardized electrical stimulation and for early prognostication in this population.

**Methods:**

This prospective observational study recruited post-CA comatose patients in Xuanwu Hospital, Capital Medical University (Beijing, China) between January 2016 and June 2023. EEG reactivity to electrical or traditional pain stimulation was randomly performed via visual and quantitative analysis. Neurological outcome within 6 months was dichotomized as good (Cerebral Performance Categories, CPC 1–2) or poor (CPC 3–5).

**Results:**

Fifty-eight post-CA comatose patients were admitted, and 52 patients were included in the final analysis, of which 19 (36.5%) had good outcomes. EEG reactivity induced with the electrical stimulation had superior performance to the traditional pain stimulation for good outcome prediction (quantitative analysis: AUC 0.932 vs. 0.849, *p* = 0.048). When using the electrical stimulation, the AUC of EEG reactivity to predict good outcome by visual analysis was 0.838, increasing to 0.932 by quantitative analysis (*p* = 0.039). Comparing to the traditional pain stimulation by visual analysis, the AUC of EEG reactivity for good prognostication by the electrical stimulation with quantitative analysis was significantly improved (0.932 vs. 0.770, *p* = 0.004).

**Conclusions:**

EEG reactivity induced by the standardized electrical stimulation in combination with quantitative analysis is a promising formula for post-CA comatose patients, with increased predictive accuracy.

## Introduction

Cardiac arrest (CA) is a critical condition, with a worldwide annual incidence of 30–97 individuals per 100,000 population [1, 2]. A majority of patients resuscitated after CA are initially comatose after the return of spontaneous circulation (ROSC) because of hypoxic-ischemic brain injury [3–6]. Withdrawal of life-sustaining therapies (WLST) may be performed following the prognostication of poor neurological outcome [7, 8]. Therefore, an early and accurate prediction of patients’ outcomes is essential to avoid inappropriate WLST. However, the established prognostication markers, such as clinical examination, neurophysiological tests and biochemical markers still show variable accuracy and are unable to provide a clear prognosis for a considerable proportion of patients [9–14].

EEG is widely used as a prognostic tool in comatose patients [15–17]. And the EEG reactivity is referred as any change in amplitude or frequency following the application of external stimulation [18]. For comatose patients, the presence of EEG reactivity to the external stimulation has been confirmed as a favorable prognostic factor [19, 20]. There is a consensus statement on EEG reactivity in comatose patients after CA; however, its application is limited by variability of stimulation types and subjectivity of EEG interpretation, which relies mainly on visual analysis (VA) of EEG signals and leaves a considerable part of coma patients after CA with uncertain prognosis [21, 22].

In routine clinical practice, EEG reactivity is mainly assessed by auditory (shouting or clapping), somatosensory (painful pressure to the nail bed or supraorbital nerve), or visual (passively eye opening) inputs. These stimuli are difficult to be standardized and their intensities and durations have interindividual differences in clinical settings, which may decrease the accuracy on EEG reactivity [23]. Additionally, the accurate interpretation of EEG reactivity induced by visual assessment limited its generalizability [24]. While, quantitative EEG analysis overcomes the challenge of subjectivity of EEG reactivity interpretation [25]. For avoiding subjectivity of stimulation types on EEG reactivity, we tried to devise a quantifiable electrical stimulation, and our previous results indicate that electrical stimulation has better performance than traditional pain stimulation [26].

On the above grounds, we hypothesized that EEG reactivity to electrical stimulation plus quantitative analysis (QA) of EEG reactivity may be an ideal procedure for the early prediction of post-CA patients’ outcomes. Therefore, this study aimed to evaluate the prognostic value of EEG reactivity using the standardized electrical stimulation and a quantitative EEG analysis method in post-CA comatose patients. Electrical stimulation was compared to pain stimulation with VA and PA separately.

## Methods

### Study design and participants

**Fig. 1:**
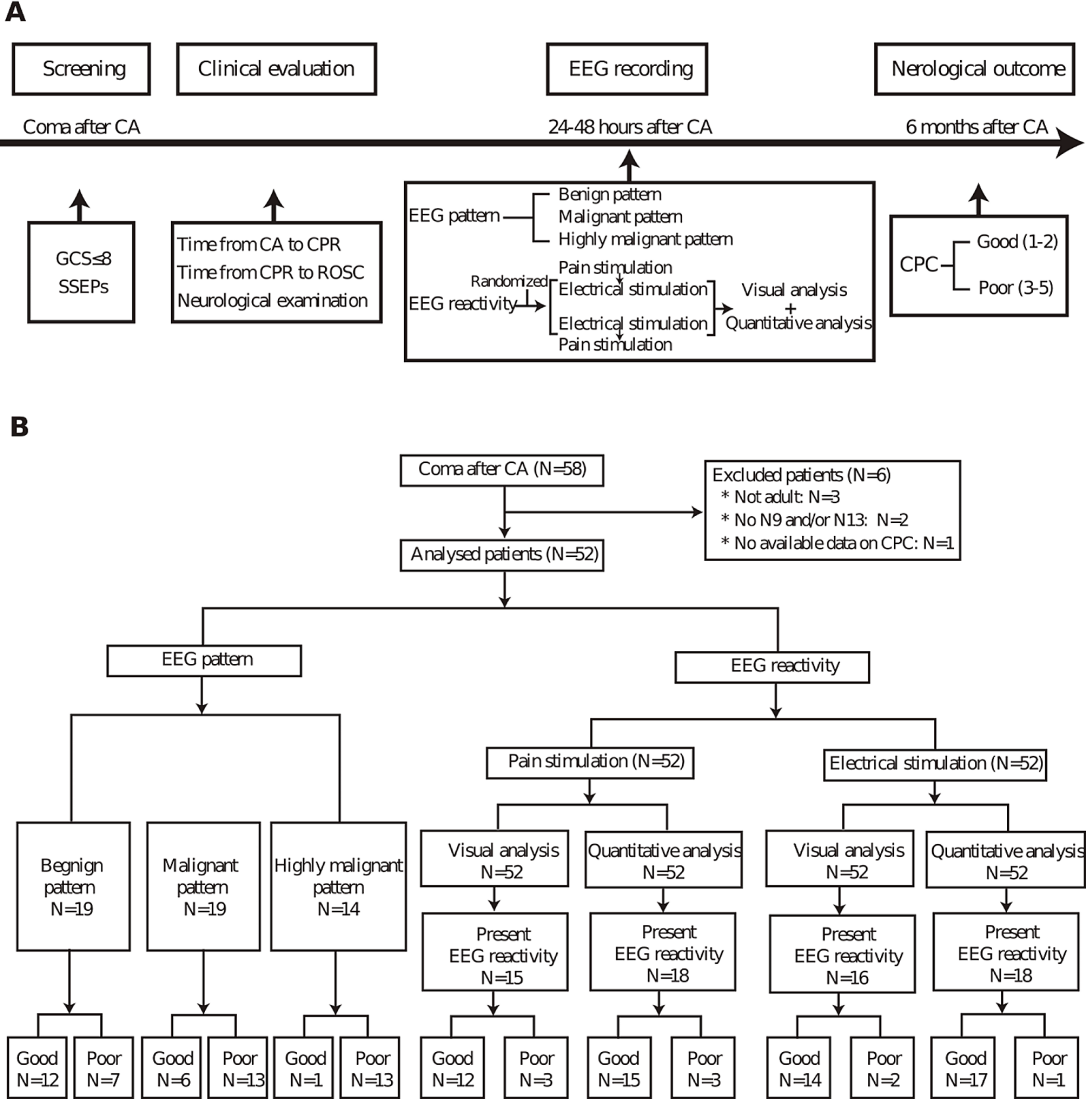
The study overview. **(A)** Summary of the study procedure. Post-cardiac arrest (CA) comatose patients were enrolled and underwent clinical evaluation. EEG was performed 24–48 h after CA. EEG reactivity was randomly assigned to the two stimulation types and evaluated separately using visual and quantitative analysis. Neurological outcome was followed as the best score on the Cerebral Performance Categories (CPC) scale within the first 6 months after CA. (**B)** Flowchart of available post-CA comatose patients for inclusion and EEG assessments

This was a single-center prospective study, and the whole study procedure was showed in Fig. 1A. We consecutively enrolled all patients with coma (Glasgow coma scale [GCS] ≤ 8) [27] after resuscitation from CA in Xuanwu Hospital, Capital Medical University between January 2016 and June 2023. We excluded patients without N9 and/or N13 on somatosensory evoked potentials (SSEPs), both are indications of the somatosensory stimulus can not be transmitted to the cerebrum. Patients with a high risk of death based on comorbidity or with prominent artifacts in EEG were also excluded.

### Procedures

Eligible post-CA comatose patients promptly underwent targeted temperature management (TTM) as a standard practice [13, 28]. Briefly, induced hypothermia was executed intravascularly or by surface cooling devices with a target core body temperature of 34–35℃ for 24 h. Analgesia and sedation were conducted with a standardized sedation-analgesia protocol by using an intravenous infusion of sauteralgyl (loading dose of 1 mg/kg, followed by a continuous infusion at a rate of 25-45 mg/hour) and midazolam (loading dose of 0.1 mg/kg, followed by a continuous infusion at a rate of 2-6 mg/hour) if necessary. Neuromuscular blockade (rocuronium) (loading dose of 0.6 mg/kg, followed by a continuous infusion at a rate of 0.3-0.6 mg/hour) might be administered intravenously in case of shivering. Rewarming (< 0.1℃/hour) was achieved passively and sedation was decreased and withdrawn at normothermia.

Neurological examinations on patients including GCS and brainstem reflexes were performed at least once daily by a neurologist at 24 to 72 h after their CA. The best evaluations on GCS and brainstem reflexes (pupillary reflex, corneal reflex, oculocephalic reflex, and cough reflex) were considered for analysis in this study [9, 13]. The following clinical variables were also collected: age, sex, site of CA event, time from CA to cardiopulmonary resuscitation (CPR), and time from CPR to ROSC.

### EEG

EEG data was recorded at bedside within 24–48 h after CA by a portable 32-channel digital EEG system (DAVINCI-SAM, Micromed, Italy). The recording electrodes, including Fp1, Fp2, F3, F4, F7, F8, C3, C4, T3, T4, T5, T6, P3, P4, O1, and O2, were placed according to the international 10–20 system. Fz electrode was used to receive stimulation signals synchronously.

EEG reactivity was assessed using two different stimuli, traditional pain stimulation (nail bed pressure) and electrical stimulation. The protocol consisted of a fixed set of traditional pain stimulation and electrical stimulation separately. Traditional pain stimulus was first performed for 5-second on the left hand and applied twice at an interval of 5-minute. Then the right hand was given pain stimulus. At least 5-minute after the end of pain stimulation, electrical stimulus was performed on the left median nerve with 5 Hz square-wave pulses lasting 2-second and applied twice with an interval of 5-minute. Then we repeated it on the right median nerve with the same frequency, strength, and duration. The stimulation would be discontinued if an individual patient showed clinical signs of arousal to the stimuli. The electrical stimulation was carried out with a stimulator of the electromyography/evoked potential machine (Nicolet Viking IV, Nicolet, Madison, WI, USA). To ensure that each subject received abundant stimulation, SSEPs were tested. The stimulus intensity that sufficiently produces a thumb twitch (0.5–1 cm) was also recorded. The range of electrical stimulus conformed to local ethical requirements. Two types of stimuli were administered in random order. If electrical stimulation was performed first, the traditional pain stimulation would be performed late. The specific ways of two stimuli were the same as described above.

***VA***: The standard definition of EEG reactivity through VA was a change in cerebral EEG activity to stimulation. This may include change in voltage or frequency, including attenuation of activity [18, 29]. Appearance of artifacts from muscle or eye blink was not considered as reactive EEG. EEG reactivity was assessed offline by two certified neurophysiologists (certified by China National Brain Injury Evaluation Quality Control Center) separately who were blinded to the clinical outcomes, and they could change the filters, signal gain, and montages of the EEG. The result of EEG reactivity assessment was dichotomized as “reactive or non-reactive”. We did not define specific values for the changes in the frequency and/or amplitude of EEG reactivity. The inconsistent results would be solved through discussion by the two neurophysiologists.

***QA***: QA was performed by EEG signal analysts who were blinded to the clinical outcomes and VA results [30]. The EEGLAB was used to perform a time-frequency analysis of the average reference montage. Firstly, a 0.5–30 Hz bandpass digital filter was used to attenuate frequency artifacts. Noise caused by eye movement was removed by using the independent component analysis algorithm. Then, potential bad channel was screened and preprocessed. After that, the EEG data were referenced to an average reference montage and 60-second-long EEG clips were extracted for each stimulation during EEG reactivity testing. The clips were divided into a pre-stimulation epoch (30-second) as the baseline and a post-stimulation epoch (30-second) after the onset of external stimulation. Secondly, the spectral power of the EEG clip was estimated. Processed time-series data were transformed into the frequency domain by a 1,024-point fast Fourier transform with Welch’s method. Specifically, EEG data was analyzed with a 512-point moving window with a 256-point overlap. Windowed data were extended to 1,024-point by zero-padding to calculate power spectra, yielding an estimation of the power spectra (0.5–30 Hz) with a 0.25 Hz frequency resolution. Finally, each spectral power of the 30-second pre-stimulation epoch and post-stimulation epoch was computed separately and averaged by all channels into a total power value. The absolute difference of spectral power between pre-stimulation and post-stimulation mean power values was used as the measure of EEG reactivity to external stimulation. The change ratio of mean power values was calculated as: $EEG \text{r}\text{e}\text{a}\text{c}\text{t}\text{i}\text{v}\text{i}\text{t}\text{y}=\frac{\left|{Power}_{post-stimulation}-{Power}_{pre-stimulation}\right|}{{Power}_{pre-stimulation}}$. EEG reactivity was measured in a normalized value, which was the ratio of power change to the stimulation. The definition of reactive EEG was a change ratio of mean power before and after stimulation ≥ 0.1 [31]. The same procedure was performed for the two types of stimuli.

In addition, EEG patterns were classified into highly malignant patterns, malignant patterns, and benign patterns according to the validated critical care EEG criteria as defined by the American Clinical Neurophysiology Society [29, 32]. Highly malignant patterns included suppressed background (< 10µV) with continuous periodic discharges, suppressed background (< 10µV) without discharges and burst-suppression background (with or without discharges) (attenuation/suppression alternating with higher voltage activity, with 50–99% of the record consisting of attenuation). Malignant patterns comprised abundant periodic discharges (> 50% of recording), abundant rhythmic spike wave (> 50% of recording), unequivocal electrographic seizure (at least one), discontinuous background with suppression periods (attenuation/suppression alternating with higher voltage activity, with 10–49% of the record consisting of attenuation or suppression), unreactive EEG (absence of background reactivity or only stimulus-induced discharges), and low voltage background (< 20µV). Benign patterns were considered as absence of any above mentioned features.

### Outcome measure

Neurological outcome was assessed by a blinded neurologist and classified as the best score on the Cerebral Performance Categories (CPC) scale within the first 6 months after CA. Good outcome was defined as CPC 1–2 (good or moderate cerebral impairment) and poor outcome as CPC 3–5 (severe cerebral impairment, vegetative state, or death) [33].

### Statistical analysis

SPSS statistical software (version 27.0) was used for all statistical analyses. We performed two-tailed t-tests for normally distributed continuous variables and chi-squared tests for confirmatory variables. A Mann-Whitney U test was performed if the variables were not normally distributed. The sensitivity, specificity, positive predictive value (PPV), and negative predictive value (NPV) of EEG reactivity were calculated, including the 95% confidence interval (CI). We used the area under the receiver operating characteristic curve (AUC) for good outcome prediction. *P* < 0.05 was considered statistically significant. A sample size of 48 was calculated to achieve 80% power to detect a difference of no less than 10% between methods based on our previous study at a significance level of two-sided 0.05 [26, 31].

## Results

### Baseline characteristics

This study screened 58 post-CA comatose patients. Of them, six were excluded for different reasons with three of less than 18-year-old, two without N9 and/or N13, and one due to lack of CPC assessment (Fig. 1B). Fifty-two subjects were included in the final analysis. Their characteristics are summarized in Table 1. Nineteen of 52 (36.5%) had good outcomes within the 6-month follow-up. There were no significant differences in age, sex, and initial GCS between the group with good outcome and that with poor outcome. The differences between the two groups in GCS motor response, brainstem reflexes, the time from CA to CPR, the time from CPR to ROSC, N20, EEG patterns, and EEG reactivity reached significance (Table 1). Patients’ status at time of EEG recording is also detailed in Table 1. There were 44 patients (84.6%) with ongoing hypothermia and 47 patients (90.4%) with ongoing sedation. Thirty-six patients (69.2%) had the best evaluations on clinical examination at time of EEG recording.

**Table 1:** Patient characteristics in relation to outcome within 6 months

Characteristic	Good outcome (CPC 1–2; *N* = 19)	Poor outcome (CPC 3–5; *N* = 33)	*P*
Age (years), mean (range)	51.2 (18–72)	53.6 (21–78)	0.713
Female, n (%)	10 (52.6)	18 (54.6)	0.894
Site of CA event (in-hospital/out-hospital)	4/15	4/29	0.443
Rhythm, n (%)			0.965
Ventricular fibrillation	3 (15.8)	4 (12.1)	
Ventricular tachycardia without pulse	2 (10.5)	3 (9.1)	
Asystole	11 (57.9)	21 (63.6)	
Pulseless electrical activity	3 (15.8)	5 (15.2)	
Etiology of CA, n (%)			0.911
Cardiac^†^	11 (57.9)	21 (63.6)	
Respiratory	6 (31.6)	9 (27.3)	
Others	2 (10.5)	3 (9.1)	
Treatment during CA, n (%)			
Defibrillation	5 (26.3)	7 (21.2)	0.739
Adrenaline	17 (89.5)	31 (93.3)	0.617
GCS, mean (range)	5 (3–8)	4 (3–8)	0.106
Glasgow motor response, range/>2, n (%)	1–3/9 (47.4)	1–3/7 (21.2)	0.164/0.049*
Brainstem reflexes, n (%)			
Pupillary reflex	12 (63.2)	10 (30.3)	0.021*
Corneal reflex	13 (68.4)	9 (27.3)	0.004*
Oculocephalic reflex	13 (68.4)	11 (33.3)	0.015*
Cough reflex	15 (79.0)	15 (45.5)	0.019*
Time from CA to CPR > 5 min, n (%)	7 (36.8)	24 (72.7)	0.011*
Time from CPR to ROSC > 20 min, n (%)	6 (31.6)	24 (72.7)	0.004*
SSEPs before EEG			
Bilateral absence of N20, n (%)	5 (26.3)	20 (60.6)	0.017*
Timing of EEG after admission (hours), median (range)	42 (24–48)	40 (24–47)	0.832
Status at time of EEG recording			
Ongoing hypothermia	16 (84.2)	28 (84.8)	1.0
Ongoing sedation	17 (89.5)	30 (90.9)	1.0
Best evaluations on clinical examination	13 (68.4)	23 (69.7)	0.723
EEG reactivity to pain stimulation, n (%)			< 0.001*
Present (VA/QA)	12 (63.2)/15 (79.0)	3 (9.1)/3 (9.1)	
Absent (VA/QA)	7 (36.8)/4 (21.1)	30 (90.9)/30 (90.9)	
EEG reactivity to electrical stimulation, n (%)			< 0.001*
Present (VA/QA)	14 (73.7)/17 (89.5)	2 (6.1)/1 (3.0)	
Absent (VA/QA)	5 (26.3)/2 (10.5)	31 (93.9)/32 (97.0)	
EEG patterns, n (%)			0.004*
Benign pattern	12 (63.2)	7 (21.2)	
Malignant pattern	6 (31.6)	13 (39.4)	
Highly malignant pattern	1 (5.3)	13 (39.4)	
Electrical stimulus intensity (mA), mean (range)	18.9 (14–36)	19.2 (15–38)	0.891
Drugs for TTM after ICU admission, n (%)			0.921
Midazolam	19 (100.0)	32 (97.0)	
Sauteralgyl	15 (79.0)	28 (84.9)	
Rocuronium	13 (68.4)	20 (60.6)	

GCS Glasgow coma scale, CPC cerebral performance categories, CA cardiac arrest, CPR cardiopulmonary resuscitation, ROSC return of spontaneous circulation, SSEPs somatosensory evoked potentials, VA visual analysis, PA quantitative analysis, TTM targeted temperature management

^†^Cardiac causes include myocardial infarction, arrhythmia, and heart failure

*p < 0.05

### Prognostic value of clinical variables and SSEPs

The AUC revealed that GCS motor response (> 2) (AUC 0.631, 95% CI 0.468 to 0.793), shorter time from CA to CPR (AUC 0.679, 95% CI 0.524 to 0.835), and shorter time from CPR to ROSC (AUC 0.706, 95% CI 0.555 to 0.857) were associated with good outcome (Table 2). Chi-squared tests revealed that presence of brainstem reflexes (pupillary reflex, corneal reflex, oculocephalic reflex and cough reflex) and N20 were associated with good outcome (*p* < 0.05).

**Table 2:** Prognostic value of clinical variables, SSEPs, and EEG for prediction of good outcome

Characteristic	AUC (95% CI)	Sensitivity (%, 95% CI)	Specificity (%, 95% CI)	PPV (%, 95% CI)	NPV (%, 95% CI)
GCS motor response	0.631 (0.468–0.793)	78.8 (61.1–91.0)	47.4 (24.4–71.1)	72.2 (54.8–85.8)	56.2 (29.9–80.2)
Time from CA to CPR	0.679 (0.524–0.835)	72.7 (54.5–86.7)	63.2 (38.4–83.7)	77.4 (58.9–90.4)	57.1 (34.0-78.2)
Time form CPR to ROSC	0.706 (0.555–0.857)	72.7 (54.5–86.7)	68.4 (43.4–87.4)	80.0 (61.4–92.3)	59.1 (36.4–79.3)
SSEPs	0.671 (0.519–0.824)	60.6 (42.1–77.1)	73.7 (48.8–90.9)	80.0 (59.3–93.2)	51.9 (31.9–71.3)
EEG pattern	0.762 (0.629–0.894)	78.8 (61.1–91.0)	63.2 (38.4–83.7)	78.8 (66.8–87.3)	63.2 (44.9–78.3)
EEG reactivity to pain stimulation					
VA	0.770 (0.648–0.892)	63.2 (38.4–83.7)	90.9 (75.7–98.1)	80.0 (53.3–92.5)	81.1 (70.2–88.6)
QA	0.849 (0.743–0.956)	79.0 (54.4–94.0)	90.9 (75.7–98.1)	83.3 (62.4–93.8)	88.2 (75.7–94.8)
EEG reactivity to electrical stimulation					
VA	0.838 (0.728–0.948)	73.7 (48.8–90.9)	93.9 (79.8–99.3)	87.5 (64.0-96.5)	86.1 (74.4–93.0)
QA	0.932 (0.855–0.983)	89.5 (66.9–98.7)	97.0 (84.2–99.9)	94.4 (71.0-99.2)	91.1 (81.2–98.4)

AUC area under the receiver operating characteristic curve, PPV predictive value, NPV negative predictive value, CI confidence interval, GCS Glasgow coma scale, CA cardiac arrest, CPR cardiopulmonary resuscitation, ROSC return of spontaneous circulation, SSEPs somatosensory evoked potentials, VA visual analysis, QA quantitative analysis

### Prognostic value of EEG pattern

In this study, 36.5% (19/52) of patients had benign EEG patterns, 36.5% (19/52) had malignant patterns, and 26.9% (14/52) had highly malignant patterns. The proportion of patients with good outcome was higher in patients with benign patterns (12/19) than those with malignant (6/19) or highly malignant (1/19) patterns (63.2% vs. 31.6% vs. 5.3%, *p* = 0.004). The EEG pattern had a superior predictive value (AUC 0.762, 95% CI 0.629 to 0.894) over clinical predictors (Table 2).

### Prognostic value of EEG reactivity

The value of EEG reactivity in predicting good outcome was summarized in Table 2.

EEG reactivity induced with electrical stimulation demonstrated superior performance compared to pain stimulation for good outcome prediction by QA (AUC 0.932 for electrical stimulation vs. 0.849 for pain stimulation, *p* = 0.048). The predicting difference with VA did not reach significance (AUC 0.838 for electrical stimulation vs. 0.770 for pain stimulation, *p* = 0.084). When using electrical stimulation, the difference in AUC for good prognostication between VA and QA reached significance (0.838 vs. 0.932, *p* = 0.039). Compared with pain stimulation by VA, the AUC for good prognostication by electrical stimulation with QA was significantly improved (0.932 vs. 0.770, *p* = 0.004).

EEG reactivity induced with electrical with QA had significantly superior performance over EEG patterns in predicting good outcome (AUC: electrical stimulation 0.932 vs. EEG pattern 0.762, *p* = 0.002). The AUC of EEG reactivity induced with electrical stimulation or pain stimulation with VA and pain stimulation with QA were superior to EEG patterns but did not reach significance (electrical stimulation and VA 0.838 vs. EEG pattern 0.762, *p* = 0.132; pain stimulation and VA 0.770 vs. EEG pattern 0.762, *p* = 0.841; pain stimulation and QA 0.849 vs. EEG pattern 0.762, *p* = 0.078).

**Fig. 2:**
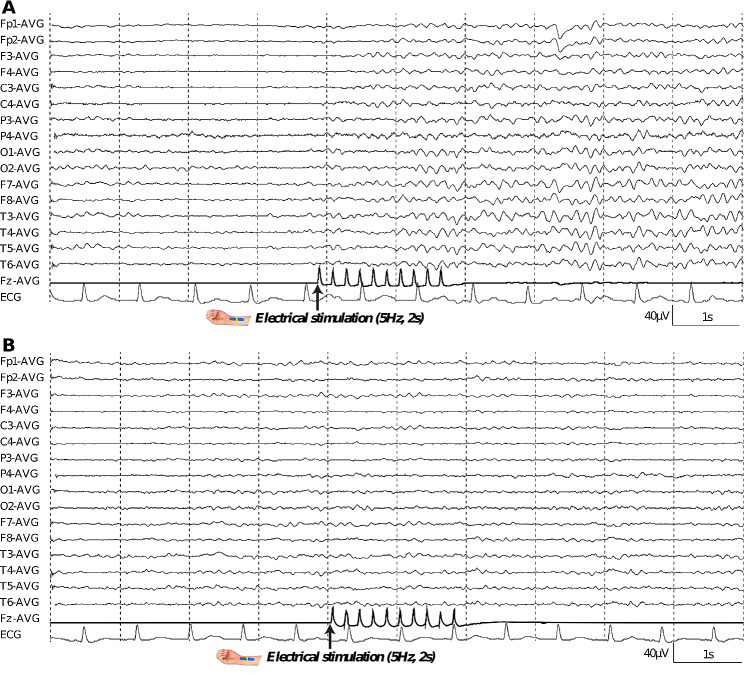
Examples of EEG reactivity using electrical stimulation through visual analysis in two cases. **(A)** Example of a present reactivity case, 40s years old, Cerebral Performance Categories (CPC) 1 within 6 months follow-up. **(B)** Example of an absent reactivity case, 50s years old, CPC 4 within 6 months follow-up

**Fig. 3:**
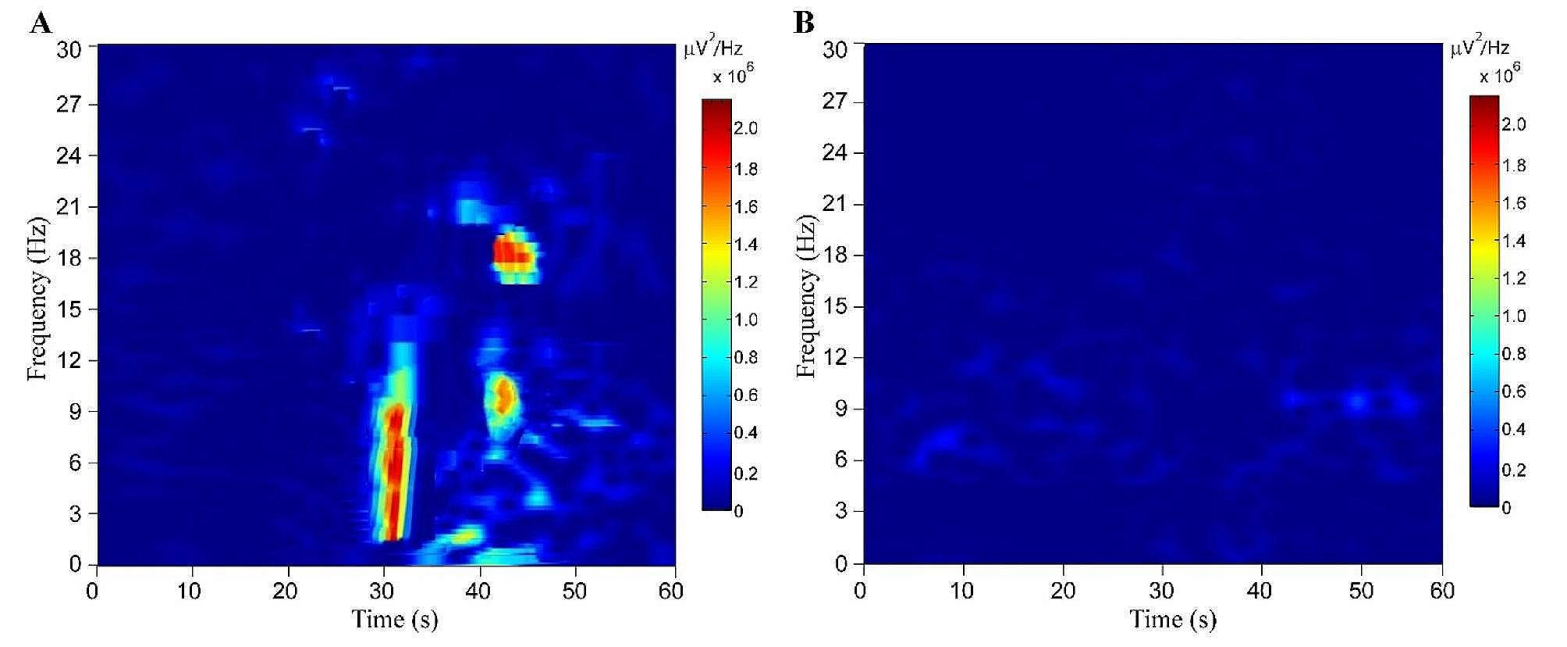
Examples of EEG reactivity through quantitative analysis in two cases. The EEG reactivity was evoked by electrical stimulation. The absolute difference of spectral power between pre- and post-stimulation mean power values was used as the measure of EEG reactivity to external stimulation. The electrical stimulus was performed on the left median nerve with 5 Hz square-wave pulses lasting 2 s. In the time-frequency plots, the x-axis denotes the time (second), and the y-axis represents the frequency (Hz). The time of 30 s indicates the onset of stimuli. The baseline is 0–30 s, and the poststimulation epoch is 30–60 s. The color bar represents energy value: blue represents a low energy value, while red represents a high energy value. (**A)** Example of a case with present EEG reactivity, 40s years old, Cerebral Performance Categories (CPC) 1 within 6 months follow-up. (**B)** Example of a case with absent EEG reactivity, 50s years old, CPC 4 within 6 months follow-up

Examples of reactive and non-reactive EEG to electrical stimulation were presented in Fig. 2. Examples of EEG reactivity by QA were shown in Fig. 3.

#### Traditional EEG reactivity: pain stimulation and VA

As shown in Table 1, reactive EEG activity by VA was present in 63.2% (12/19) of patients with good outcomes and 9.1% (3/33) of patients with poor outcomes (*p* < 0.01). Reactive EEG had a sensitivity of 63.2% (95% CI 38.4 to 83.7%), a specificity of 90.9% (95% CI 75.7 to 98.1%), a PPV of 80.0% (95% CI 53.3 to 92.5%) and an NPV of 81.1% (95% CI 70.2 to 88.6%) in predicting good outcome.

#### EEG reactivity induced with pain stimulation and QA

Reactive EEG activity by QA was present in 79.0% (15/19) of patients with good outcomes and 9.1% (3/33) of patients with poor outcomes, respectively (*p* < 0.001). QA of EEG reactivity had a sensitivity of 79.0% (95% CI 54.4 to 94.0%), a specificity of 90.9% (95% CI 75.7 to 98.1%), a PPV of 83.3% (95% CI 62.4 to 93.8%) and an NPV of 88.2% (95% CI 75.7 to 94.8%). Comparing to VA, QA had a higher sensitivity (79.0% vs. 63.2%), but had the same specificity (90.9% vs. 90.9%).

#### EEG reactivity induced with electrical stimulation: VA or QA

No noticeable artifacts were observed when performing electrical stimulation. A total of 8 patients did not have thumb movement due to neuromuscular blockade for TTM (3 in good outcome group and 5 in poor outcome group, *p* > 0.05). These 5 patients had N9 and/or N13 on SSEPs. When using VA, 14 out of 16 patients with reactive EEG had good outcomes and 31 of 36 patients with non-reactive EEG had poor outcomes. The PPV with reactive EEG had good outcome was 87.5% (95% CI 64.0 to 96.5%). The NPV with non-reactive EEG for poor outcome was 86.1% (95% CI 74.4 to 93.0%). The reactive EEG had a sensitivity of 73.7% (95% CI 48.8 to 90.9%) and a specificity of 93.9% (95% CI 79.8 to 99.3%) to predict good outcome.

When using QA, reactive EEG activity was present in 89.5% (17/19) of patients with good outcomes and 3.0% (1/33) of patients with poor outcomes (*p* < 0.001). The reactive EEG had a sensitivity of 89.5% (95% CI 66.9 to 98.7%), a specificity of 97.0% (95% CI 84.2 to 99.9%), a PPV of 94.4% (95% CI 71.0 to 99.2%) and an NPV of 91.1% (95% CI 81.2 to 98.4%).

## Discussion

We studied the prognostic value of EEG reactivity to electrical stimulation with QA in predicting coma outcome after CA. We demonstrate that EEG reactivity via the standardized electrical stimulation has superior performance to traditional pain stimulation in predicting good outcome 6 months later. In addition, the quantitative EEG analysis is superior to VA, and the combination of electrical stimulation with QA has the highest predictive value, with an AUC of 0.932. Our approach provides an objective and valuable way to perform EEG reactivity for early prognostication in post-CA coma.

Our study showed that the sensitivity and specificity of EEG reactivity for outcome prediction with pain stimulation was in line with previous findings [19, 34–36]. We obtained the best accuracy with a sensitivity of 89.5% and specificity of 97.0% by electrical stimulation and QA, which was higher than electrical stimulation with VA (73.7% sensitivity and 93.9% specificity), pain stimulation with VA (79.0% sensitivity and 88.2% specificity), and pain stimulation with QA (63.2% sensitivity and 90.9% specificity). And, 35.4% of the post-CA comatose patients had good outcomes, which was consistent with previous studies [37–39]. Despite the improvement of post-CA care, many patients those may have good outcome if their continuation of life-sustaining therapies, will die after WLST following a prognostication of poor neurological outcome [8]. Since a subset of post-CA patients are expected to have good outcome, an accurate prognostication in these patients is essential for the continuation of intensive life support measures and avoiding premature WLST.

Previous studies have shown that EEG reactivity is a predictor in comatose patients. However, the stimulation protocols and predictive value of different stimulus types between studies change widely. External stimulations often include auditory stimuli by calling the patient’s name, somatosensory stimuli by pressing on the nail bed, and visual stimuli by passive eye-opening or light exposure [18]. These conventional stimuli are difficult to be standardized and may vary among different performers, which can be avoided by quantified stimulation. In our study, electrical stimulation can be easily quantified with electrical square-wave pulses. EEG reactivity to electrical stimuli can improve prognostication of coma outcome. It is semi-automatic and based on existing techniques, which can provide additional insights for the outcome prediction.

Our results demonstrated QA was comparable or even superior to VA with the same stimulation. Several studies also indicated that QA of EEG is at least as good as VA, even with varied methodologies [25, 30, 31]. QA of EEG have the following significant advantages over VA. First, it is not subjected to inter-rater variability. Second, it is not restricted to highly trained personnel and is easily popularized. Moreover, it can be fully automated and is less time-consuming. Therefore, this method is more suitable for predicting coma outcome after CA in the clinical routine.

EEG reactivity represents the neural activity along the afferent somatosensory pathways through the ascending reticular activating system to the cortex. The electrical stimulation of the median nerve is a mechanical stimulus within a certain stimulus intensity. Low intensity electrical sensation is conducted through a different neural transduction pathway than pain stimulation [40]. When the threshold of stimulus intensity is exceeded, pain sensation may also occur [41]. To avoid the influence of conductive pathway interruption, we excluded two patients without N9 and/or N13. Though it is still unclear how best to perform EEG reactivity testing, pain stimulation (nail bed pressure) is one of the most common methods evoking EEG reactivity [23, 25, 30, 34, 35]. Therefore, we chose this traditional pain stimulation as a comparison.

SSEP was performed before EEG for screening reason to exclude patients without N9 and/or N13 (indication of the somatosensory stimulus cannot be transmitted to the cerebrum) in this study. Our results showed that 26% of patients with good outcome had bilaterally absent N20, which was high than previous reports [42, 43]. To achieve good predicting value, the recommended monitoring time for SSEP is at least more than 24 h after ROSC, or even after rewarming [44, 45]. We did not repeat SSEP testing at the recommended time. The early testing of SSEP might result in our decreased predicting value. Our results also showed that 3.4% (2/58) of patients did not have N9 and/or N13. Therefore, there is no need for extensive screening SSEP before EEG. SSEP can be performed at the recommended time, and we suggest further clarification on whether there is N9 and/or N13 in these patients without EEG reactivity to avoid the risk of potential false results.

The major strengths of this study were the prospective design for collecting clinical information, resuscitation details, and neurological examinations in all patients. Additionally, we separately compared the prediction value of clinical evaluation, absence of N20 with the SSEPs, EEG pattern, and EEG reactivity to the pain or electrical stimulation under visual assessment and QA. Another advantage of this study was that the use of electrical stimulation could be easily quantified with electrical square-wave pulses, further ensuring the generalizability of our findings. Moreover, the follow-up was done until 6-month after CA and the best CPC was analyzed.

This study also had some limitations. First, video recording was not included when performing EEG reactivity testing. The lack of video EEG recording might make it more challenging to distinguish artifacts from EEG signals, potentially bringing uncertainty to the interpretation of EEG reactivity. In clinical practice, video EEG recordings could improve reliable interpretation. Second, we only performed once EEG testing within 24–48 h after CA, which may be affected by sedation and hypothermia. There is still controversy over this potential effect of sedation and hypothermia on EEG. Several studies found that the early EEG findings showed good accuracy for the prediction of outcome and were not significantly affected by hypothermia as well as sedation drugs [26, 35, 46]. Third, reactivity is suggested to be assessed when reproducible after three sets of stimulus application [22]. Our study did not perform and compare other types of stimuli, which may restrict the generalization of our method. Fourth, our method itself (30 s without artifacts) may limit its generalization in the intensive care unit. Fifth, the group size was relatively small and future studies are needed to confirm our findings in a large sample.

## Conclusion

We showed the standardized electrical stimulation and QA of EEG to predict good outcome from coma in patients after CA. This finding confirmed that EEG reactivity is a significant favorable prognostic factor for neurological outcomes in post-CA comatose patients. Our work also showed that electrical stimulation and QA can further improve the prognostic accuracy for early prognostication of coma outcome. To achieve ideal prognostic value, EEG reactivity testing is performed by the standardized electrical stimulation with QA should be recommended to use in the clinical routine.

## Data Availability

The data and materials are available from the corresponding author upon reasonable request.

## Abbreviations

AUC: Area under the receiver operating characteristic curve
CA: Cardiac arrest
CPC: Cerebral Performance Categories
CI: Confidence interval
CPR: Cardiopulmonary resuscitation
GCS: Glasgow coma scale
NPV: Negative predictive value
PPV: Predictive value
ROSC: Return of spontaneous circulation
SSEPs: Somatosensory evoked potentials
TTM: Targeted temperature management
WLST: Withdrawal of life-sustaining therapies
